# INFRAFRONTIER—providing mutant mouse resources as research tools for the international scientific community

**DOI:** 10.1093/nar/gku1193

**Published:** 2014-11-20

**Authors:** INFRAFRONTIER Consortium

## Abstract

The laboratory mouse is a key model organism to investigate mechanism and therapeutics of human disease. The number of targeted genetic mouse models of disease is growing rapidly due to high-throughput production strategies employed by the International Mouse Phenotyping Consortium (IMPC) and the development of new, more efficient genome engineering techniques such as CRISPR based systems. We have previously described the European Mouse Mutant Archive (EMMA) resource and how this international infrastructure provides archiving and distribution worldwide for mutant mouse strains. EMMA has since evolved into INFRAFRONTIER (http://www.infrafrontier.eu), the pan-European research infrastructure for the systemic phenotyping, archiving and distribution of mouse disease models. Here we describe new features including improved search for mouse strains, support for new embryonic stem cell resources, access to training materials via a comprehensive knowledgebase and the promotion of innovative analytical and diagnostic techniques.

## INTRODUCTION

For over a century, mouse strains have been used as models to study the genetic, environment and pharmacological components of human disease (1). The applicability of mouse models to translational research is evident in the growing number of mutant mouse resources that are becoming available. To date, over 10 000 mouse genes have had mutant alleles described in the literature (2). The International Mouse Phenotyping Consortium (IMPC) is generating a knockout mouse strain for every protein-coding gene and characterising their phenotypes in a broad-based phenotyping pipeline (3,4). In conjunction, new technologies are greatly increasing the efficiency and speed by which mutant mouse strains are being generated. Especially promising are techniques using CRISPR/CAS9 endonucleases that target genes without the need for embryonic stem (ES) cell culturing and could cut production time for mutant mouse strains in half or more (5,6).

Mouse repositories play an important role in promoting and distributing the resources produced by both large- and small-scale mouse projects. In a research era of limited funding and ethical concern about unnecessary experimental animal production, mouse repositories reduce costs and duplication by providing high quality mouse strains with supporting documentation so that researchers do not need to generate strains of their own. Repositories also frequently provide training and protocols for best practice in the breeding and experimental use of mouse models. The European Mouse Mutant Archive (EMMA) is one such repository that coordinates archiving and distribution of mice across 16 national partners and provides up to date information about strain availability. Since the resource was described in the 2010 NAR database issue (7), EMMA has merged with the wider INFRAFRONTIER project and supports standardized mouse phenotyping services in Europe to ensure results are robust and reproducible. The merger of the two projects has the expanded goals of:

1. Providing access to mouse models, data and scientific platforms and services.
2. Determining genotype–phenotype interactions through cutting-edge analytical and diagnostic methodology in the INFRAFRONTIER mouse clinics.
3. Archiving and distribution of mouse strains.

Here we present new features and improved architecture supporting these goals.

## NEW INFRAFRONTIER WEB PORTAL HIGHLIGHTS AVAILABLE SERVICES

The new web portal was built based on user feedback and reflects the diverse and dynamic nature of the project by being organized into several distinct sections. A news section highlights recent events that are of relevance to the INRAFRONTIER community and are updated frequently. Resources and services offered by the project are highlighted in boxes containing icons and links that lead users to detailed pages. The ability to search and browse available mouse strains is also offered on the home page. At the top of the page, four drop down menus expand into an extensive hierarchy that allows users to drill down to features described below.

### Enhanced search for mutant mouse strains

**Figure 1.**
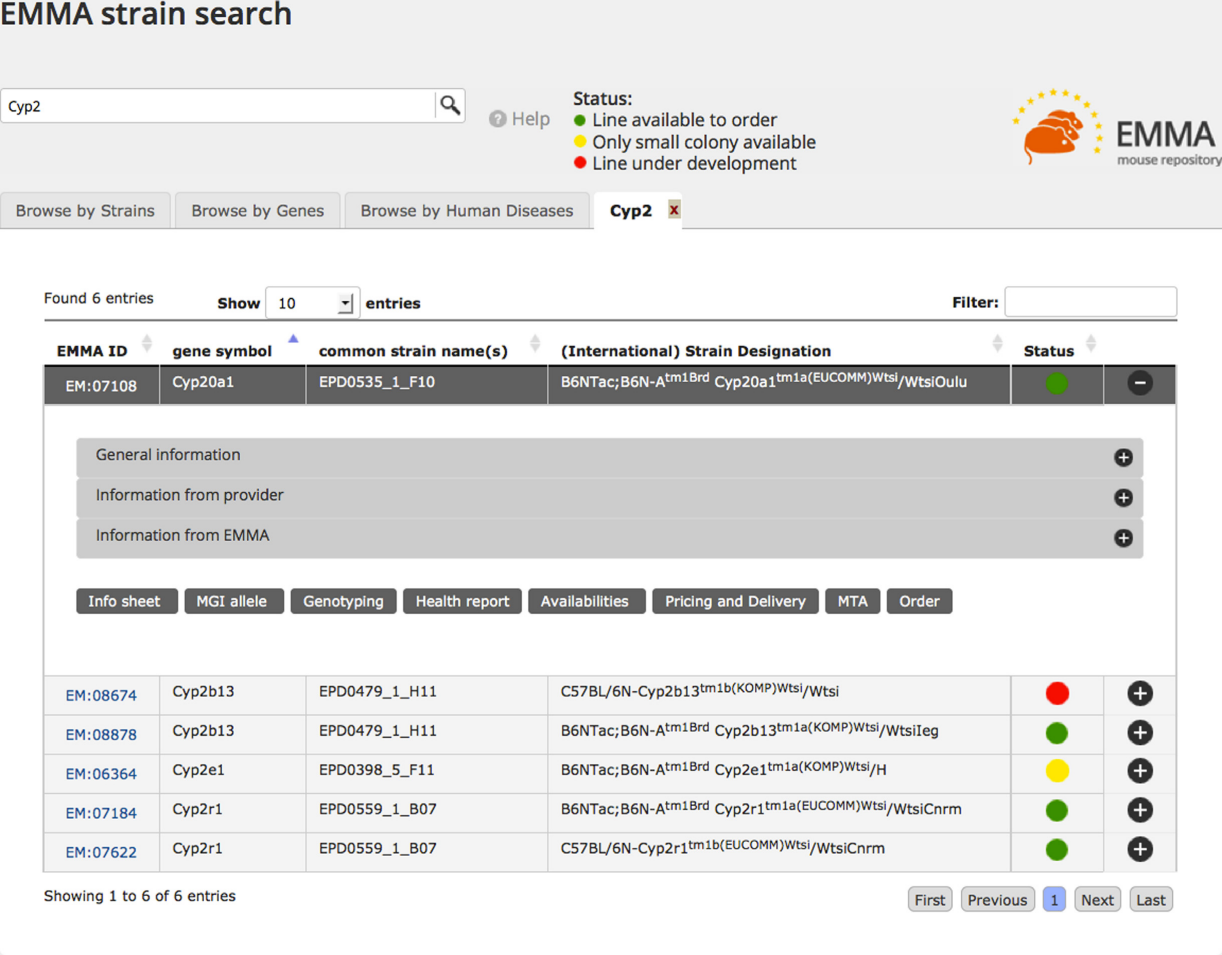
INFRAFRONTIER strain search. A simplified grid summing up available mouse strains is returned based on lexical matching. Rows are expanded when the plus icon is selected, giving summaries on a wide range of data available for a strain.

One of our highest priorities is to enhance searching or browsing for strains (Figure 1). This page underwent extensive redesign to present only the minimum information needed for users to find appropriate mouse strains and retains the EMMA logo to provide continuity from the previous project. User testing and online surveys found that the three biggest determinants are the (i) gene mutated in a given strain, (ii) strain names with the allele(s) propagated in the strain and (iii) whether the strain is available to order. The results table provides this critical data and is also flexible, returning a disease as described in the Online Mendelian Inheritance of Man (OMIM) with mouse models as manually associated by the Mouse Genome Informatics (MGI) at Jackson Laboratory based on authors’ assertions in the scientific literature (2). Each search creates a new tab on the results table so that users can compare results from multiple searches. Tabs also contain interfaces to browse by mouse strain based on disease models, gene symbol and by allele type. As availability status of strains is determined by a number of factors, we use the simple visualisation of the familiar red, yellow, green traffic light icons that represent ‘under development’, ‘only a small colony is available’ and ‘lines available to order’. A user can click on the plus icon in a result row and a new menu is presented that includes an extensive array of secondary information related to a strain including husbandry information, material transfer agreement (MTA) or licensing documents (if any) and pricing (Figure 1).

As of August 2014, the INFRAFRONTIER resource contains over 4178 submitted strains from 21 countries including 2052 lines generated by high-throughput projects such as the IMPC. Of these strains, over 1465 (3656 requests from 42 countries for 1465 individual strains) have been requested from researchers worldwide. While the majority of orders come from within Europe, a significant percentage of orders come from North America (32%) and Asia (15%).

### Online strain submission tool

**Figure 2.**
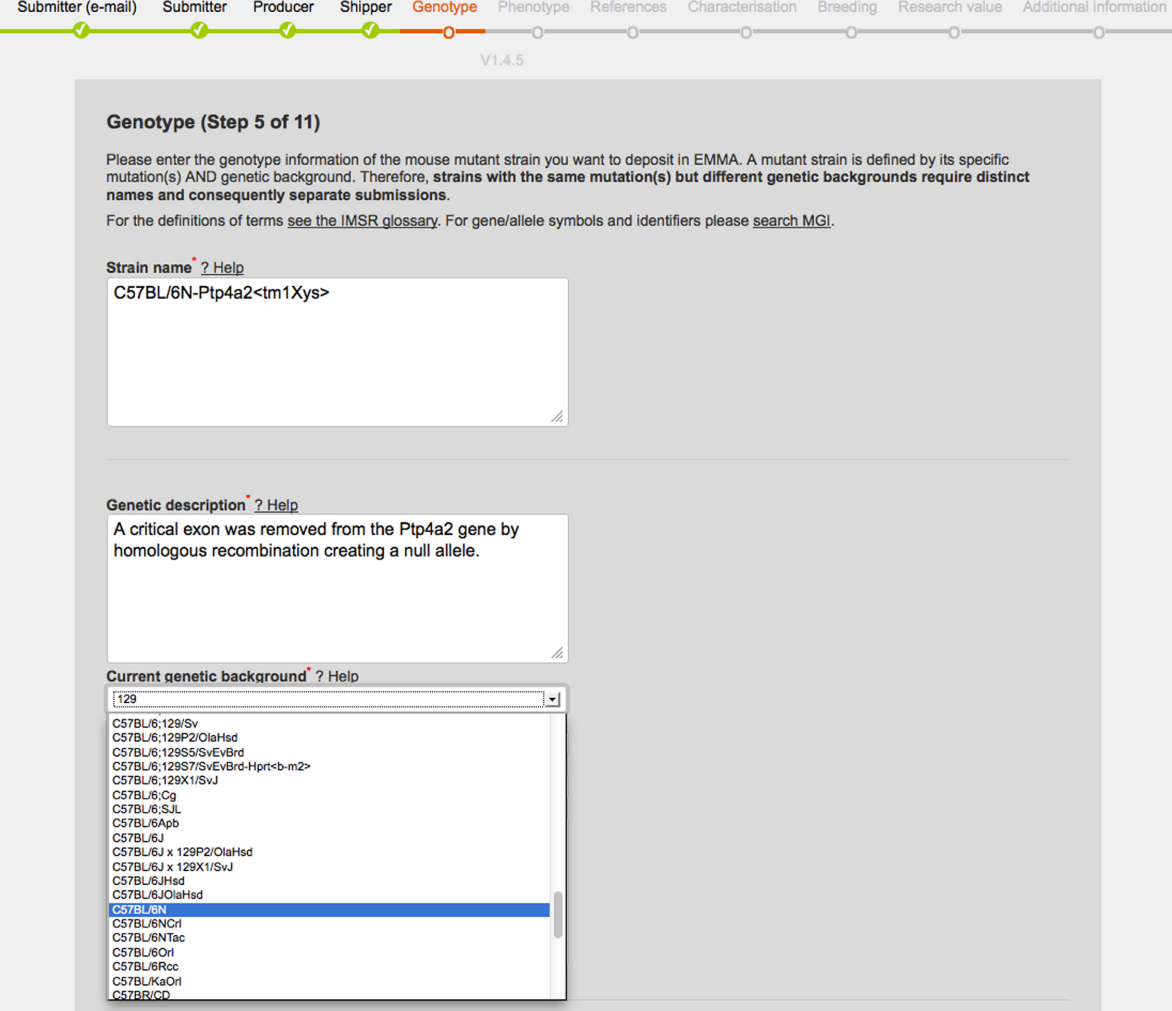
Online strain submission tool. An online tool guides users through a series of steps to upload data about a strain they wish to deposit in the EMMA resource. Screenshot depicts the step where genotype information is entered. The use of pull down menus reduces user errors and curation burden.

About half of the strains available from the INFRAFRONTIER resource come from smaller scale projects and individual labs that deposit strains to make available to others. As the information needed is quite extensive and broad ranging, an online wizard has been developed that guides users through an 11-step process (Figure 2). An email address is requested in step 1 to act as a unique identifier that can recall previous submissions that were started but not completed. Specific tools are incorporated into the wizard including the ability to navigate to previous steps, pull down menus of commonly used ES cell lines and genetic background provided from MGI, and automated metadata annotation when a PubMed or OMIM ID is given. Upon successful completion, a PDF summarising the information given is emailed back to the submitter.

### The Netherlands Cancer Institute embryonic stem cells archive

Another new service that INFRAFRONTIER provides is the distribution of mutant mouse embryonic stem cells (ESC) derived from validated genetically engineered mouse models (GEMM-ESCs) of cancer created by the Netherlands Cancer Institute (NKI) (8). Breeding of cancer mouse models is difficult as they typically involve multiple dominant alleles that do not necessarily co-segregate and thus need to be genotyped at each step of breeding. The NKI GEMM-ESCs addresses this challenge by providing ESC that carry common oncogenic alleles and include additional genomic landing sites for easy insertion of new oncogenic or tumour-suppressor transgenes. From a dedicated section of the INFRAFRONTIER portal, users can order ES cell clones, download MTAs, find maps of the mutant alleles and obtain protocols for culturing the cells and inserting transgenes.

### Promotion of training courses

INFRAFRONTIER through its partners offers a wide range of state-of-the-art training opportunities including popular cryopreservation courses and best practices for broad-based mouse phenotyping. The INFRAFRONTIER web portal promotes upcoming training events through dedicated web pages, news bulletins and related protocols being stored in the knowledgebase.

### Free production and phenotyping of mouse strains

Free mouse strain production and broad-based phenotyping are provided to eligible partners in a series of competitive calls. Information and applications are available from the INFRAFRONTIER portal and outreach is performed via an INFRAFRONTIER contact list as well as other sources.

### Axenic services

Mice derived by aseptic techniques and reared and maintained under germ-free conditions are important research tools for immunological and microbiome studies. This service was previously offered as part of EMMA and now includes rich documentation on the facility supporting this service, key references and a request form.

### Rich, navigable knowledgebase

Through its diverse activities, INFRAFRONTIER has developed a rich knowledgebase with five broad categories—protocols, bibliography, workshops and policy documents. An easy to navigate hierarchy with the ability to drill down to specific documents and other media is included as part of the knowledgebase. As these documents are core to the training activities, they are frequently updated and represent the state of the art. Presentations from INFRAFRONTIER workshops are also available allowing users to stay informed of diverse subject manners that are the focus of these workshops. A Drupal framework for the portal provides a Wiki-like interface to facilitate addition of content by non-technical personnel so that users may be quickly informed about news, events and have access to the latest protocols within the knowledgebase.

### INTEROPERABILITY AND SUSTAINABILITY

High-throughput mouse production by IMPC centres is a quickly growing source of mouse strains. Extensive development has gone into an automated curation procedure that uses IMPC tracking information to populate the database when a strain is to be distributed by INFRAFRONTIER. The data collected include the ES cell clone used to generate the mouse strain, the mutant allele, the genetic background and the distribution centre. From this information, a customized algorithm incorporates the allele and genetic background of contributing strains to automatically generate strain nomenclature. This algorithm is frequently reviewed and modified to reflect procedural chances in mouse strain production.

Extensive expert curation takes place to correct, standardize and complete the original data provided by the external submitters, taking advantage of new and efficient curation interfaces. These interfaces use official nomenclature for gene, allele, transgene, background names and symbols and help identify when new gene or allele nomenclature is needed. The curation is based on the application of rules and guidelines established by the International Committee on Standardized Genetic Nomenclature for Mice (http://www.informatics.jax.org/mgihome/nomen) for the initial assignment and periodic review and update of strain nomenclature.

Our effort to assign proper nomenclature to mouse alleles and strains facilitates coordination with other resources. Mechanisms have been introduced to ensure gene and allele nomenclature is up to date by obtaining the latest nomenclature from MGI. Preliminary strain designations made by INFRAFRONTIER curators are submitted, weekly reviewed and subsequently promoted by the International Mouse Strain Resource (9). In a similar manner, the IMPC portal generates links from gene pages to corresponding INFRAFRONTIER mutant strains based on shared identifiers. Standardized strain nomenclature also allows users to find phenotype associations for a given strain when it is described in the literature. This facilitates comparisons of phenotypes for related strains studied by multiple resources to ensure associations are robust and reproducible. For example, Nbeal2 mutant mice distributed by INFRAFRONTIER (strain B6NTac;B6N-A^tm1Brd^ Nbeal2^tm1a(EUCOMM)Wtsi^/WtsiCnbc) are characterized by the IMPC as having increased platelet size and decreased circulating platelet cell numbers (http://www.mousephenotype.org/data/genes/MGI:2448554). This is similar to a published report about a different mutant strain that describes Nbeal2 deficient mice as having defective platelet development and function, and being a strong candidate model for the human disease grey platelet syndrome (GPS) (10). Because both strains have been annotated with the Mammalian Phenotype ontology (11,12), a researcher can be reasonably confident that the Nbeal2 deficient strain from INFRAFRONTIER will recapitulate the phenotypes and be a model for GPS. In fact, there are several on-going efforts to systematically score models based on shared phenotype features with human disease patients (13,14). Such approaches will better inform users about which strains they need and IMPC phenotypes and disease modelling scores maybe included in future updates of the INFRAFRONTIER portal.

Interoperability promotes uptake and use of INFRAFRONTIER resources, a critical factor in sustainability. Another factor is the stability of funding. The INFRAFRONTIER GmbH was created to coordinate the transnational activities of INFRAFRONTIER. With the involvement and financial commitment of the members states that have founded the GmbH, a new level of financial sustainability and planning reliability is in place, ensuring that INFRAFRONTIER will be able to continue its mission of serving the worldwide scientific community.

## CONCLUSIONS

INFRAFRONTIER provides many resources supporting translational research including the archiving and distribution of mouse disease models, a rich knowledgebase, and training of a new generation of researchers in the use and production of mouse models of disease. Emerging technologies promise to greatly increase gene–disease associations and the ability to make relevant mouse disease models. INFRAFRONTIER is in a unique position to scale up in parallel with this increased output and allow scientists to find the resources needed to support their research for many years to come.

## APPENDIX

INFRAFRONTIER is prepared by Terrence F. Meehan, Chao-Kung Chen, Gautier Koscielny, Mike Relac, Phil Wilkinson, Paul Flicek and Helen Parkinson at the European Molecular Biology Laboratory, European Bioinformatics Institute; Ana Castro, Sabine Fessele, Ralph Steinkamp, Michael Hagn, Michael Raess and Martin Hrabé de Angelis at the Helmholtz Zentrum München GmbH; Joanna Bottomley, Ramiro Ramirez-Solis and Damian Smedley at the Wellcome Trust Sanger Institute; Simon Ball, Andrew Blake, Martin Fray, Janet Kenyon, Ann-Marie Mallon and Steve Brown at the Medical Research Council, Harwell; Marzia Massimi, Raffaele Matteoni and Glauco Tocchini-Valentini at the Consiglio Nazionale delle Ricerche, EMMA-Monterotondo Campus International Development; Yann Herault at the Institut de Génétique et de Biologie Moléculaire et Cellulaire (IGBMC), et Institut Clinique de la Souris (ICS) and PHENOMIN; George Kollias at the Biomedical Sciences Research Center Alexander Fleming; Brun Ulfhake at the Karolinska Institutet; Jocelyne Demengeot at the Instituto Gulbenkian de Ciência; Cecile Fremond at the Centre National de la Recherche Scientifique-TAAM; Fatima Bosch at the Universitat Autonoma de Barcelona; Lluis Montoliu at the Centro Nacional de Biotecnologia (CNB-CSIC) and CIBER-ER-ISCIII; Raija Soininen at the University of Oulu; Klaus Schughart at the Helmholtz Zentrum für Infektionsforschung GmbH; Cord Brakebusch at the University of Copenhagen; Radislav Sedlacek at the Institute of Molecular Genetics of the ASCR; Thomas Rülicke at the Veterinärmedizinische Universität Wien, Institute of Laboratory Animal Science and Biomodels Austria; Colin McKerlie at the Toronto Centre for Phenogenomics Inc; Bernard Malissen at the Centre d'Immunophénomique, INSERM; Fuad Iraqi at the Tel Aviv University; Jos Jonkers at the Netherlands Cancer Institute; Holger Russig at the TSE Systems GmbH; and Danny Huylebroeck at the Lab. Molecular Biology (Celgen), Department of Development and Regeneration, Stem Cell Institute, KU Leuven and Department of Cell Biology, ErasmusMC.
